# Extinction Dynamics of the Foot-and-Mouth Disease Virus Carrier State Under Natural Conditions

**DOI:** 10.3389/fvets.2020.00276

**Published:** 2020-05-20

**Authors:** Miranda R. Bertram, Shankar Yadav, Carolina Stenfeldt, Amy Delgado, Jonathan Arzt

**Affiliations:** ^1^Foreign Animal Disease Research Unit, Plum Island Animal Disease Center, ARS, USDA, Orient Point, NY, United States; ^2^Oak Ridge Institute for Science and Education, PIADC Research Participation Program, Oak Ridge, TN, United States; ^3^Monitoring and Modeling, Center for Epidemiology and Animal Health, APHIS, USDA, Fort Collins, CO, United States; ^4^Department of Diagnostic Medicine/Pathobiology, Kansas State University, Manhattan, KS, United States

**Keywords:** foot-and-mouth disease, FMDV, persistent infection, extinction dynamics, Vietnam, India, meta-analysis, carrier

## Abstract

Foot-and-mouth disease (FMD) is one of the most economically important livestock diseases worldwide. Following the clinical phase of FMD, a large proportion of ruminants remain persistently infected for extended periods. Although extinction of this carrier state occurs continuously at the animal and population levels, studies vary widely in their estimates of the duration of persistent infection. There is a need for robust statistical models to capture the dynamics of persistent infection for the sake of guiding FMD control and trade policies. The goal of the current study was to develop and assess statistical models to describe the extinction of FMD virus (FMDV) persistent infection using data from primary longitudinal studies of naturally infected cattle and Asian buffalo in Vietnam and India. Specifically, accelerated failure time (AFT) models and generalized linear mixed models (GLMM) were developed to predict the probability of persistent infection in seropositive animals and identified carriers at the individual animal level at sequential time points after outbreaks. The primary studies were analyzed by country and combined using an individual-participant data meta-analysis approach. The models estimated similar trends in the duration of persistent infection for the study/species groups included in the analyses, however the significance of the trends differed between the models. The overall probabilities of persistent infection were similar as predicted by the AFT and GLMM models: 6 months: 99% (AFT) /80% (GLMM), 12 months: 51% (AFT) /32% (GLMM), 18 months: 6% (AFT) /5% (GLMM), 24 months: 0.8% (AFT) /0.6% (GLMM). These models utilizing diverse and robust data sets predict higher probabilities of persistence than previously published, suggesting greater endurance of carriers subsequent to an outbreak. This study demonstrates the utility of statistical models to investigate the dynamics of persistent infection and the importance of large datasets, which can be achieved by combining data from several smaller studies in meta-analyses. Results of this study enhance current knowledge of the FMDV carrier state and may inform policy decisions regarding FMDV persistent infection.

## Introduction

Foot-and-mouth disease virus (FMDV; *Aphthovirus, Picornaviridae*) is the causative agent of foot-and-mouth disease (FMD), one of the most economically important diseases of livestock worldwide. Classical FMD is characterized by fever, loss of appetite, and formation of characteristic vesicles on feet, udders, and in the oral cavity (1, 2). Although mortality is usually low, the high morbidity has an important economic impact due to decreased production, regional quarantine practices, and trade restrictions (3, 4). The existence of prolonged asymptomatic persistent infection (carrier state) in ruminants has practical implications in FMDV-endemic regions that are distinct from management practices in regions striving to regain FMD-free status after an outbreak (5). Appropriate practices for management of carriers have not been established in either context.

Subsequent to acute infection, a substantial proportion of infected ruminants become persistently infected, which has traditionally been defined by detection of FMDV in oropharyngeal fluid (OPF) 28 days or more after infection (6, 7). However, more recent studies have indicated that persistently infected animals can be identified as early as 10 days post-infection (8). Vaccination with a homologous virus strain protects against clinical disease, but does not prevent subclinical or persistent infection (8–10). The virus persists in the epithelium of the nasopharynx (8, 11) or associated lymphoid tissue (12) of cattle and buffalo. Transmission from persistently infected cattle to naïve animals via direct contact has not been demonstrated (10, 13, 14), however deposition of oropharyngeal fluid from persistently infected cattle into the nasopharynx of naïve cattle has been demonstrated to cause disease (15). Transmission via direct contact in the persistent phase has only been demonstrated to occur from African Cape buffalo (*Syncerus caffer*) (16, 17), and the role of persistently infected animals in FMDV epidemiology remains unclear. However, concerns over the potential risk of transmission from persistently infected animals have prompted authorities to implement trade restrictions for animals and animal products for extended periods following FMD outbreaks and from FMD-endemic regions (18).

FMDV is reported to persist for up to 2 years in cattle (14, 19, 20), 5–12 months in sheep and goats (21), and up to 5 years in African buffalo (22). However, the virus is cleared from carriers at variable times by mechanisms which have been described (23, 24). The rate of decrease in the proportion of persistently infected animals has been reported as 0.03–0.11 per month (13, 14, 19). A meta-analysis of experimental studies reported that most infected cattle clear the infection within 6 months (13); however recent field studies indicated that approximately half of infected cattle remain persistently infected 12 months after infection (19), and some cattle maintain persistent infection for more than 24 months (14). The variability among distinct studies and analytical approaches impedes development of effective control measures to account for FMDV persistent infection. Most importantly there is a need for robust methods to describe and predict the duration of persistent infection at the individual and population levels.

Recent longitudinal field studies have utilized survival analysis to describe the dynamics of extinction of persistent infection in cattle under natural endemic conditions (14, 19). These studies demonstrated gradual clearing of the infection over time at the population level; however, they did not predict the probability of persistent infection at specific time points in the study populations. A recent analytical approach proposed defining extinction of persistent infection based on a probability function to better reflect the dynamic state of persistent infection (25). These authors used cross-sectional data to develop a statistical model to estimate the probability of persistent infection based on an animal's age, whether the animal had antibodies against FMDV, and the time since the most recent outbreak in the herd. This approach to predicting the probability of the presence of persistently infected animals in a herd at a defined time(s) following an outbreak may be beneficial for developing FMD control policies. However, the very low probability (0.7%) of persistent infection across all animals more than 12 months after an outbreak reported in that study is inconsistent with the data from recent longitudinal field studies of FMDV-infected animals (14, 19).

Longitudinal studies offer the advantage of directly observing the dynamics of persistent infection in individuals over time. Disadvantages of longitudinal studies are that they are highly labor-intensive, time-consuming, expensive, and logistically challenging under field conditions in endemic regions. In contrast, cross-sectional studies often have larger sample sizes and can be completed more rapidly and economically than longitudinal studies. However, it is unclear whether cross-sectional data is appropriate for modeling the dynamics of persistent infection. Alternatively, meta-analyses could be used to mitigate some of the challenges of small sample sizes by combining data across several longitudinal studies (26). Meta-analysis approaches incorporating data from multiple studies have the additional advantage of incorporating diverse field conditions (viral strain, host factors, husbandry, environmental factors) into a more holistic output.

The goals of the current study were to assess the utility of two distinct statistical models for predicting the probability of persistent FMDV infection post-outbreak at the individual animal level, and to compare different analytical methods to assess extinction of the carrier state. The current study incorporated and analyzed three primary longitudinal studies of FMDV persistent infection in Vietnam and India using both generalized linear mixed models and accelerated failure time models to predict the probability of persistent infection in cattle and Asian buffalo (*Bubalus bubalis*) at various times following an FMD outbreak. Additionally, data from all three studies were combined using an individual participant data meta-analysis approach (26) to further assess the dynamics of persistent infection across the three study populations. Results of this study will help to inform FMD surveillance and control efforts in Vietnam, India, and other FMD-endemic countries as well as FMD-free countries, and will help to inform policy decisions concerning FMDV persistent infection.

## Methods

To qualify for inclusion in these analyses, studies had to take place following a natural outbreak in an FMD-endemic country, OPF samples had to be collected from the same individual animals at least twice, and sampling had to occur 28 days or more post-outbreak. Additionally, raw data had to be available for each animal. Three studies from our laboratory met the inclusion criteria—one in Vietnam and two in India. The primary datasets incorporated in the analyses herein were derived within the scope of long-term, longitudinal projects on endemic FMD in India and Vietnam between 2010 and 2015 (14, 19, 27–29). The included studies are described below. For the current study, persistent infection was defined as the detection of FMDV RNA in OPF.

### Ethics Approval

The work described herein was performed by federal staff of the Department of Animal Health, Ministry of Agriculture, and Rural Development, Government of Vietnam or the Directorate of Foot and Mouth Disease, Indian Council of Agricultural Research, Ministry of Agriculture, Government of India. The work occurred and the animals were maintained within facilities that were owned, maintained, or overseen by these divisions of the federal governments; thus, no permits or approvals were required. All cases described herein occurred spontaneously in domestic cattle or buffalo with no experimentation, inoculation, or treatment of live animals.

### Vietnam

#### Study Description

**Figure 1:**
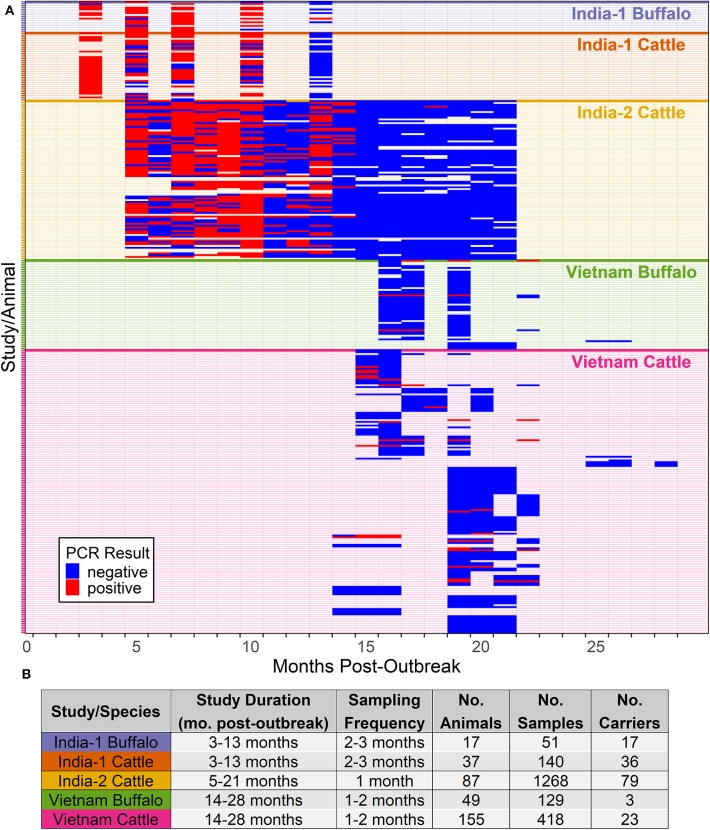
FMDV RNA detection in oropharyngeal fluid by rRT-PCR. Data are presented from two studies in India and one in Vietnam, and include samples from cattle and Asian buffalo. **(A)** Each row represents one animal and the grouping of rows is colored according to study/species group. Study/species group is labeled on the right side of the figure and colored corresponding to the study/species in the table in **(B)**. Columns represent sampling times (months post-outbreak), and samples are represented by red (positive) and blue (negative) bars. **(B)** A summary of the data is provided in the table.

Cattle and Asian buffalo were sampled as part of a targeted surveillance study in areas with a recent history of FMD outbreaks in Long An and Son La provinces in Vietnam as previously described (27, 29). Briefly, serum samples were collected in March 2012, and subsequently oropharyngeal fluid (OPF) samples were collected using a probang cup (30) from 323 animals that were seropositive for FMDV anti-NSP antibodies using a 3ABC ELISA kit (PrioCheckR, Prionics, Netherlands). OPF samples were collected every 1–2 months between April—October 2012 for up to 4 samples per animal (Figure 1). OPF samples were analyzed by real-time reverse transcription PCR (rRT-PCR) targeting the 3D region of the FMDV genome as previously described (31). Briefly, RNA was extracted using the MagMax Viral RNA Isolation Kit (Ambion), and extracted RNA was subjected to rRT-PCR using a previously described probe (32) and primers (33). As previously reported, 10.8% of seropositive animals were carriers, based on FMDV RNA detection in OPF, and beef cattle were more likely to be carriers than buffalo or dairy cattle (27). A subset of 155 cattle and 49 buffalo from 93 herds for which the owner reported the animal's history of clinical FMD (yes or no) were analyzed in the current study. Vaccination status was reported for ~70% of animals, a majority (97%) of which had been previously vaccinated. However, the date of vaccination was not available for most animals, and the effect of vaccination on the duration of persistent infection could not be evaluated in this study.

#### FMD Outbreak History

For animals for which the owner reported a history of clinical FMD, the year of infection was reported by the owner. The date (month and year) of the outbreak which occurred in the Commune of the herd during the reported year of infection was retrieved from records of the Department of Animal Health (DAH), Vietnam. The midpoint of the month was used as the outbreak date in subsequent analyses. For animals for which the owner reported no history of clinical FMD, the outbreak date reported for other animals in the herd was used as the outbreak date. If no previous FMD infection was reported in the herd, the date of the most recent outbreak in the Commune prior to sampling was used as the outbreak date. For the purposes of this study, it was assumed that animals were infected during the reported outbreak and no reintroduction or subclinical circulation of the virus occurred on farms included in the study. This assumption was based upon documentation by farm-level questionnaire and by reviewing official records of the Department of Animal Health that no cases of FMD were observed or reported in the intervening period. The virus strain was not identified for all animals; however, all carrier animals from which sequence data were obtained were infected with strains from lineage O/ME-SA/PanAsia (27).

### India

#### Study Descriptions

Cattle and Asian buffalo were sampled following two independent outbreaks in large dairy herds in India as previously described (19, 28, 34). Despite biosecurity practices in place, both premises had vulnerabilities to FMDV incursion, including unvaccinated cattle in the surrounding areas, and personnel moving between the farm and their personal livestock. The source of the virus was not identified in either outbreak.

Briefly, one study (India-1) investigated persistent infection in cattle and Asian buffalo following an FMD outbreak on a large dairy farm in India, which occurred in January 2014 (28, 34). Animals were vaccinated 3–4 times a year with a trivalent (A, O, Asia-1) vaccine, and had most recently been vaccinated ~50 days prior to the outbreak. A convenience sample of 37 cattle and 17 buffalo, identified as carriers based on FMDV RNA detection in OPF, were sampled at 2–3 months intervals from 3–13 months post-outbreak (Figure 1). FMDV RNA was detected by rRT-PCR as described for Vietnam samples. The sampling included animals that were clinically or subclinically infected during the outbreak. As previously reported, all study animals were seropositive for FMDV anti-NSP antibodies by r3AB3 I-ELISA (35). Additionally, all study animals were persistently infected 3 months post-outbreak, and 7–17% of cattle and buffalo, respectively, remained persistently infected 13 months post-outbreak. The duration of persistent infection was not significantly different between clinically and subclinically affected animals, nor between cattle and buffalo (34).

The second study (India-2) investigated persistent infection in dairy cattle on one management unit in India following an FMD outbreak which occurred in October 2013 (19). Animals were vaccinated twice yearly with a trivalent (A, O, Asia-1) vaccine, and had most recently been vaccinated 3–4 days prior to the outbreak. A convenience sample of 47 juvenile and 31 adult cattle, identified as carriers based on FMDV RNA detection in OPF as described for Vietnam samples, were sampled monthly from 5–21 months post-outbreak (Figure 1). The sampling included animals that were clinically or subclinically affected during the recent outbreak. As previously reported, all study animals were seropositive for FMDV anti-NSP antibodies by r3AB3 I-ELISA (35). The average duration of persistent infection was 13.1 months, and all animals cleared the infection by 19 months post-outbreak. There was no significant difference in the duration of persistent infection between clinically and subclinically affected animals (19). An additional 9 animals that entered the study late were included in the current analyses.

Analyses of the duration of persistent infection have been reported previously for each of the India studies (19, 28, 34). Additionally, both outbreaks were caused by the same strain of FMDV (O/ME-SA/Ind2001d), and the studies had similar study designs. Therefore, the two India studies were combined in the current analyses by country as well as in the meta-analyses of all three studies, and estimates were produced for each study/species group. For the purposes of this study, it was assumed that animals were infected during the reported outbreak and no novel incursion or subclinical circulation of the virus occurred on the farms included in the study. This assumption was based upon documentation by the herd veterinarians that no cases of FMD were observed or reported during the period of the study and the finding that all carrier viruses from which sequence data was acquired were phylogenetically closely related to the outbreak strains (34).

### Statistical Analysis

#### Accelerated Failure Time Model

For each country separately and for all three studies combined, the probability of persistent infection was investigated using interval-censored survival analysis, in which the time to event is modeled as an interval rather than an exact time. Failure to detect FMDV RNA in an OPF sample was the event of interest. The elapsed time between the outbreak date and the date of the last positive sample (rounded to the nearest month) was used as the low end of the interval, and the elapsed time between the outbreak date and the date of the next negative sample (rounded to the nearest month) was used as the high end of the interval in which the event occurred. For animals without a positive sample (i.e., left-censored), the low end of the interval was set to “NA,” and for animals that remained persistently infected throughout the study (i.e., right-censored), the high end of the interval was set to “NA,” as recommended by the software package authors. For the initial analysis, the Kaplan-Meier estimator was used to create a survival curve (data not shown). Due to small sample sizes and violations of the proportional hazards assumption, accelerated failure time (AFT) analyses were used, since they perform better than Cox proportional hazard analyses under these conditions (36, 37). To account for species differences and other study-site related variability, a combined study and species variable was created (study/species). Preliminary models were fit using the Weibull, exponential, log-logistic, and log-normal distributions, and the distribution which minimized the Akaike information criterion (AIC) was selected as the appropriate distribution for the final model. Analyses were implemented in R v3.5.3 using the *survival* base package (38). Goodness of fit of the final models was evaluated by visualization of the qq-plots of times of survival percentiles, Cox-Snell residuals, and comparison of predicted survival curves to Kaplan-Meier curves, as implemented in the *AFTtools* package (39). The final models were used to predict the duration of FMDV RNA detection at percentiles from 0.01 to 0.99 using the *predict* function in the *survival* package, and the results were subsequently used to estimate the probability of FMDV RNA detection at 6, 12, 18, and 24 months post-outbreak. Figures were created using the *ggplot2* package (40).

#### Generalized Linear Mixed Model

For each country separately and for all three studies combined, the probability of persistent infection was investigated using Generalized Linear Mixed Model (GLMM). Detection of FMDV RNA in OPF (yes/no) was the outcome variable, and the main independent variable was the elapsed time (rounded to the nearest month) between the outbreak date and the sample collection date. The combined study/species variable was also included as a fixed effect to account for variability among studies and species. Additionally, individual ID was included as a random variable to account for repeated measures on the same animals. GLMMs were built including time post-outbreak (in months) with and without the combined study and species variable, and the best fit model was selected considering the statistical and biological relevance. The model building and analyses were performed in R v3.5.2 using the *lme4* package (41). The final model equations were used to predict the probability of FMDV RNA detection in OPF at 6, 12, 18, and 24 months post-outbreak in Microsoft Excel 2019. Figures were created using the *ggplot2* package in R v3.5.2 (40).

## Results

### Observed Extinction Dynamics (All Primary Studies)

The final dataset used to investigate the dynamics of extinction of persistent infection consisted of 2,006 samples from 345 seropositive animals or identified carriers, across the 3 studies (Figures 1, **4A**). All farms included in the analyses reported no FMD cases during the timeframe of the study or within 28 days prior to the start of the study. As a result, animals from which FMDV RNA was detected in OPF during the study were considered persistently infected.

**Figure 2:**
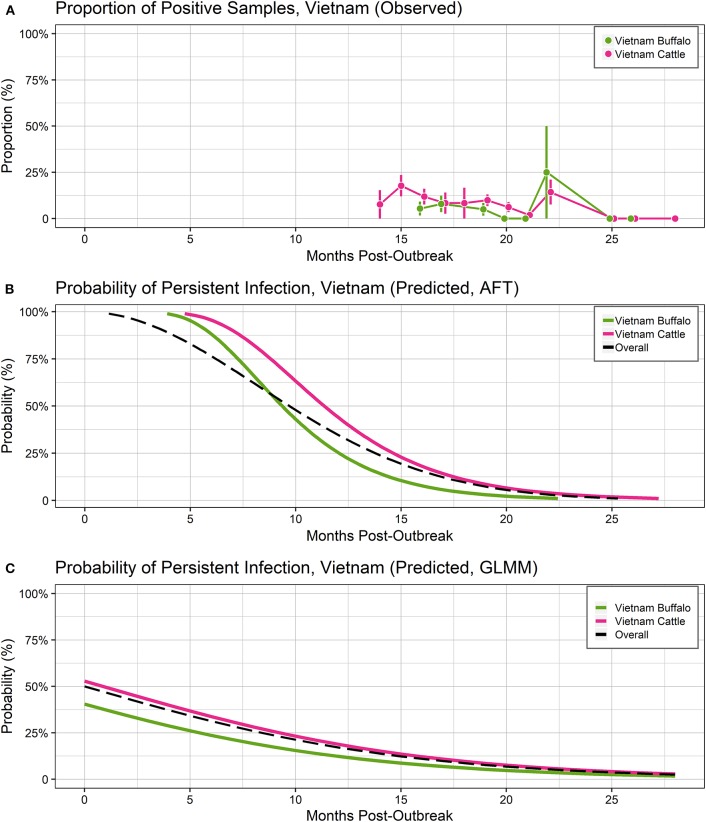
Probability of persistent infection, Vietnam data only. All study animals were seropositive for FMDV anti-NSP antibodies by 3ABC-ELISA. **(A)** Observed proportion of OPF samples positive for FMDV RNA. Mean and standard error are shown. **(B)** Predicted probability of persistent infection, AFT model. **(C)** Predicted probability of persistent infection, GLMM model.

**Figure 3:**
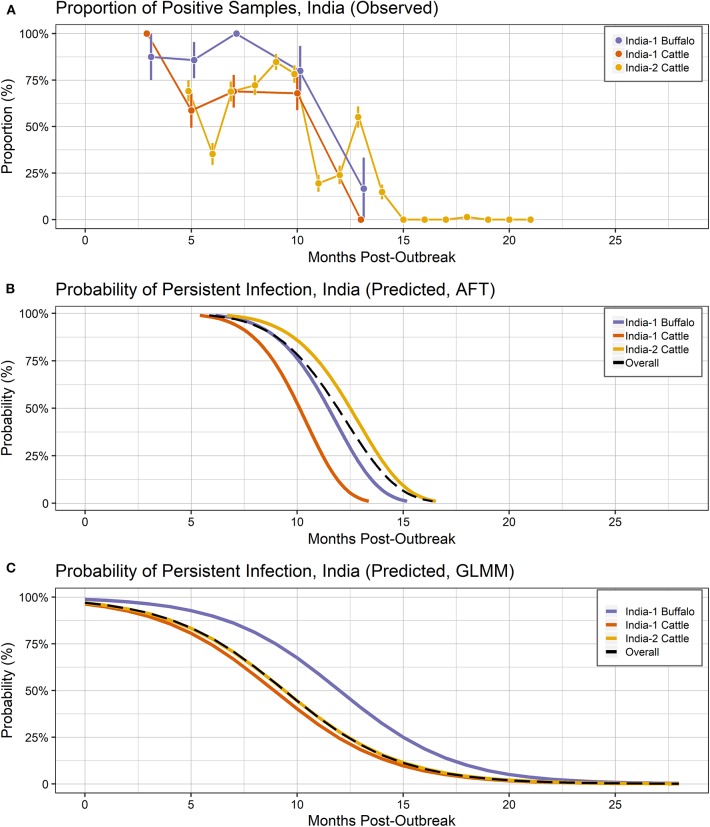
Probability of persistent infection, India data only. All study animals were seropositive for FMDV anti-NSP antibodies by r3AB3 I-ELISA. **(A)** Observed proportion of OPF samples positive for FMDV RNA. Mean and standard error are shown. **(B)** Predicted probability of persistent infection, AFT model. **(C)** Predicted probability of persistent infection, GLMM model.

In Vietnam, FMDV RNA was detected in ~8% of samples at the first sampling time, 14 months post-outbreak, which increased to 22% at 15 months post-outbreak, then gradually decreased. No FMDV RNA was detected in samples collected after 25 months post-outbreak (Figure 2A). In the India-1 study, FDMV RNA was detected in all cattle samples and ~90% of buffalo samples at the first sampling time 3 months post-outbreak, and the proportion decreased gradually until 10 months post-outbreak, with a rapid decrease between 10 and 13 months post-outbreak. Approximately 15% of buffalo samples were persistently infected at the last sample 13 months post-outbreak, whereas no FMDV RNA was detected in any cattle samples at that time (Figure 3A). In the India-2 study, FMDV RNA was detected in ~70% of cattle samples at the first sample 5 months post-outbreak, and the proportion tended to decrease until 15 months post-outbreak. No FMDV RNA was detected after 15 months post-outbreak, with the exception of one animal at 18 months post-outbreak (Figure 3A).

### Modeled Dynamics of Persistent Infection in Vietnam

#### Accelerated Failure Time Model (Vietnam)

Overall, 26/204 (12.7%) animals were persistently infected in the Vietnam study. Based on AIC, the log-normal distribution was most appropriate for the final model (AIC = 215.14), and the model assumptions were appropriate based on evaluation of goodness of fit. In the final model, the duration of FMDV RNA detection in OPF for cattle and buffalo did not differ significantly (*p* = 0.1) (Table 1).

**Table 1:** Accelerated failure time model for the duration of FMDV RNA recovery from oropharyngeal fluid following an FMD outbreak in Vietnam.

**Variable**	Coef^*^	Std. Err^**^	***P*-value**	**Time ratio**	**95% CI**
Intercept	2.24	0.16	<0.0001		
Vietnam buffalo	(ref)				
Vietnam cattle	0.19	0.12	0.1	1.21	0.96, 1.53
log(scale)	−0.98	0.20	<0.0001		

* *Coef, Model coefficient*.

** *Std. Err, standard error*.

*The model was fitted using the log-normal distribution*.

Using the AFT final model, the predicted probability of persistent infection in seropositive animals in Vietnam 6 months post-outbreak was 88% for buffalo and 95% for cattle, which decreased to 25 and 44%, respectively, 12 months post-outbreak, and <1% for buffalo and 2% for cattle at 24 months post-outbreak (Table 2, Figure 2B).

**Table 2:** Predicted probability of persistent infection following an FMD outbreak.

**Study**	**Species**	**AFT predictions**				**GLMM predictions**			
		**Months post-outbreak**				**Months post-outbreak**			
		**6**	**12**	**18**	**24**	**6**	**12**	**18**	**24**
Vietnam only	Vietnam buffalo	88.18%	25.44%	4.09%	0.61%	23.63%	12.36%	6.04%	2.84%
	Vietnam cattle	95.52%	44.15%	11.01%	2.32%	33.78%	18.86%	9.58%	4.60%
	Vietnam overall	76.48%	34.81%	9.53%	1.57%	31.30%	17.19%	8.64%	4.13%
India only	India-1 buffalo	99.15%	39.28%	0%	0%	89.97%	49.95%	9.99%	1.22%
	India-1 cattle	98.01%	11.03%	0%	0%	74.35%	24.38%	3.46%	0.40%
	India-2 cattle	99.52%	59.24%	0.03%	0%	77.63%	27.85%	4.12%	0.48%
	India overall	98.84%	48.6%	0.03%	0%	77.90%	27.69%	4.00%	0.45%
India and Vietnam	India-1 buffalo	99.5%	40.88%	2.47%	0.24%	91.05%	51.75%	10.16%	1.18%
	India-1 cattle	98.26%	16.49%	0.72%	0.07%	75.21%	24.23%	3.26%	0.35%
	India-2 cattle	99.75%	58.51%	4.9%	0.49%	77.56%	26.70%	3.70%	0.40%
	Vietnam buffalo	99.58%	45.61%	2.98%	0.29%	75.77%	24.79%	3.36%	0.36%
	Vietnam cattle	99.86%	71.08%	8.25%	0.85%	87.21%	41.82%	7.04%	0.79%
	Overall	99.23%	50.75%	5.76%	0.82%	80.38%	32.08%	5.17%	0.6%

*Predictions were made from three longitudinal studies in Vietnam and India using an accelerated failure time model (AFT) and a generalized linear mixed model (GLMM)*.

#### Generalized Linear Mixed Model (Vietnam)

Overall, FMDV RNA was detected in 46/547 (8.4%) total samples from the Vietnam study. The best fit model included time post-outbreak and the study/species variable. In the final model, the time post-outbreak was a significant variable (*p* = 0.04), and the odds of persistent infection decreased by 12% with each month post-outbreak (Table 3). Similar to the AFT model, the odds of persistent infection did not differ significantly between cattle and buffalo (*p* = 0.2).

**Table 3:** Generalized linear mixed model for the probability of FMDV RNA recovery from oropharyngeal fluid following an FMD outbreak in Vietnam.

**Variable**	Coef^*^	Std. Err^**^	***P*-value**	**Odds ratio**	**95% CI**
Intercept	−0.38	1.21	0.75		
Month post-infection	−0.13	0.06	**0.04**	**0.88**	0.78, 0.99
Vietnam buffalo	(ref)				
Vietnam cattle	0.5	0.4	0.21	1.65	0.75, 3.61

* *Coef, Model coefficient*.

** *Std. Err, standard error*.

*Significant values are indicated in bold*.

Using the GLMM final model, the predicted probability of persistent infection in seropositive animals in Vietnam 6 months post-outbreak was 23% for buffalo and 34% for cattle, which decreased to 12 and 19%, respectively, 12 months post-outbreak, and 3 and 5% at 24 months post-outbreak (Table 2, Figure 2C).

### Dynamics of Persistent Infection in India

#### Accelerated Failure Time Model (India)

Overall, 132/141 (93.6%) animals were persistently infected in the two India studies. Based on AIC, the Weibull distribution was most appropriate for the final model (AIC = 470.96), and the model assumptions were appropriate based on evaluation of goodness of fit. In the final model, the duration of FMDV RNA detection in OPF for India-1 buffalo and India-2 cattle did not differ significantly (*p* = 0.2) (Table 4). In contrast, the duration was 0.8 times shorter for India-1 cattle than for India-2 cattle, and the difference was significant (*p* < 0.0001).

**Table 4:** Accelerated failure time model for the duration of FMDV RNA recovery from oropharyngeal fluid following an FMD outbreak in India.

**Variable**	Coef^*^	Std. Err^**^	***P*-value**	**Time ratio**	**95% CI**
Intercept	2.58	0.02	<0.0001		
India-1 buffalo	−0.09	0.07	0.19	0.92	0.81, 1.04
India-1 cattle	−0.21	0.04	**<0.0001**	**0.81**	0.75, 0.87
India-2 cattle	(ref)				
log(scale)	−1.91	0.08	<0.0001		

* *Coef, Model coefficient*.

** *Std. Err, standard error*.

*The model was fitted using the Weibull distribution*.

*Significant values are indicated in bold*.

Using the AFT final model, the predicted probability of persistent infection in identified carriers in India 6 months post-outbreak was >99% for India-1 buffalo and India-2 cattle, and 98% for India-1 cattle. At 12 months post-outbreak, the probability decreased to 39% for India-1 buffalo and 59% for India-2 cattle, whereas the decrease was even greater for India-1 cattle (11% probability at 12 months post-outbreak). At 18 months post-outbreak, the probability of persistent infection was <0.05% for all groups (Table 2, Figure 3B).

#### Generalized Linear Mixed Model (India)

Overall, FMDV RNA was detected in 511/1,459 (35.0%) total samples from the two studies in India. The best fit model included time post-outbreak and the study/species variable. The odds of persistent infection decreased by 31% with each month post-outbreak (Table 5). In contrast to the AFT model, the odds of persistent infection were 2.6 times higher in India-1 buffalo compared to India-2 cattle, and the difference was significant (*p* = 0.01). Contrastingly, the odds of persistent infection were not significantly different between India-1 cattle and India-2 cattle (*p* = 0.4).

**Table 5:** Generalized linear mixed model for the probability of FMDV RNA recovery from oropharyngeal fluid following an FMD outbreak in India.

**Variable**	Coef^*^	Std. Err^**^	***P*-value**	**Odds ratio**	**95% CI**
Intercept	3.44	0.20	0.0		
Month post-infection	−0.37	0.01	**0.0**	**0.69**	0.68, 0.71
India-1 buffalo	0.95	0.37	**0.01**	**2.59**	1.25, 5.34
India-1 cattle	−0.18	0.21	0.41	0.84	0.55, 1.26
India-2 cattle	(ref)				

* *Coef, Model coefficient*.

** *Std. Err, standard error*.

*Significant values are indicated in bold*.

Using the GLMM final model, the predicted probability of persistent infection in identified carriers in India 6 months post-outbreak was 90% for India-1 buffalo, 74% for India-1 cattle, and 78% for India-2 cattle. The GLMM predicted higher probability of persistent infection at later timepoints compared to the AFT: 4–10% at 18 months post-outbreak and 0.5–1% at 24 months post-outbreak (Table 2, Figure 3C).

### Dynamics of Persistent Infection Across All Studies

In order to achieve the most robust estimates possible, the probability of persistent infection across all primary studies was modeled in a similar approach, which examined the duration of persistent infection (AFT) and the effect of time on the probability of persistent infection (GLMM). The goal of this combined meta-analysis was to benefit from the breadth of variability of study design, viral, host, and environmental factors included across the three primary studies. The study/species variable was included in all models to account for species and study-site variability. Estimates were generated for each study/species group. Estimates are reported for a target population of seropositive animals, but may be considered biased upward due to the inclusion of only identified carriers in the India studies.

#### Accelerated Failure Time Model (Combined Studies)

In total, 158/345 (45.8%) animals were carriers across the three studies. For the analysis of all primary studies combined, the log-logistic distribution was most appropriate for the final model (AIC = 722.71), and the model assumptions were appropriate based on evaluation of goodness of fit. In the final model, the duration of persistent infection was 0.8 times shorter for India-1 cattle compared to India-2 cattle (*p* < 0.0001). For all other groups, the duration of persistent infection was not significantly different from India-2 cattle (Table 6).

**Table 6:** Accelerated failure time model for the duration of FMDV RNA recovery from oropharyngeal fluid following an FMD outbreak across three studies.

**Variable**	Coef^*^	Std. Err^**^	***P*-value**	**Time ratio**	**95% CI**
Intercept	2.53	0.02	<0.0001		
India-1 buffalo	−0.09	0.08	0.26	0.92	0.79, 1.07
India-1 cattle	−0.24	0.05	**<0.0001**	**0.79**	0.71, 0.87
India-2 cattle	(ref)				
Vietnam buffalo	−0.06	0.08	0.43	0.94	0.80, 1.10
Vietnam cattle	0.07	0.04	0.08	1.07	0.99, 1.15
log(scale)	−2.10	0.08	<0.0001		

* *Coef, Model coefficient*.

** *Std. Err, standard error*.

*The model was fitted using the log-logistic distribution*.

*Significant values are indicated in bold*.

**Figure 4:**
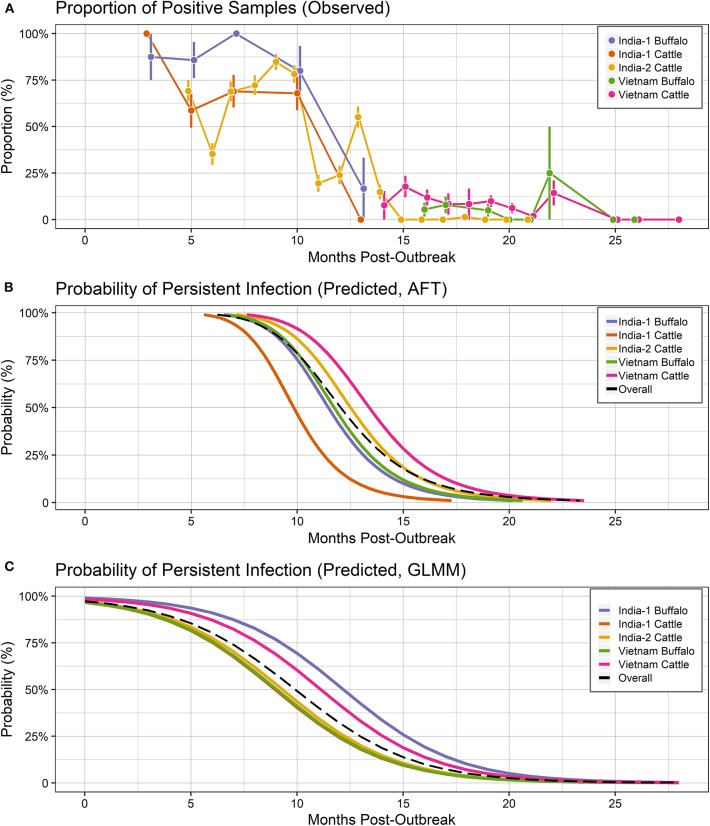
Probability of persistent infection, Vietnam and India data combined. All study animals were seropositive for FMDV anti-NSP antibodies by 3ABC-ELISA (Vietnam) or by r3AB3 I-ELISA (India). **(A)** Observed proportion of OPF samples positive for FMDV RNA. Mean and standard error are shown. **(B)** Predicted probability of persistent infection, AFT model. **(C)** Predicted probability of persistent infection, GLMM model.

For all groups combined, the overall probability of persistent infection in seropositive animals predicted using the AFT final model was 99.23% (range: 98.26–99.86%) at 6 months after an outbreak, 50.75% (range: 16.49–71.08%) at 12 months post-outbreak, 5.76% (range: 0.72 – 8.25%) at 18 months post-outbreak, and at 24 months post-outbreak was 0.82% (range: 0.07–0.85%) (Table 2, Figure 4B).

#### Generalized Linear Mixed Model (Combined Studies)

For the analysis of all studies combined, the best fit model included time post-outbreak and the study/species variable. In the final model, the odds of persistent infection decreased by 31% with each month post-outbreak (Table 7). In contrast to the AFT model, the odds of persistent infection at any given time were three times higher in India-1 buffalo and two times higher in Vietnam cattle compared to India-2 cattle (*p* = 0.02 and *p* = 0.01, respectively). Additionally, the odds of persistent infection were not significantly different for India-1 cattle compared to India-2 cattle (*p* = 0.7).

**Table 7:** Generalized linear mixed model for the probability of FMDV RNA recovery from oropharyngeal fluid following an FMD outbreak across three studies.

**Variable**	Coef^*^	Std. Err^**^	***P*-value**	**Odds ratio**	**95% CI**
Intercept	3.49	0.22	0.0		
Month post-infection	−0.38	0.02	**0.0**	**0.68**	0.66, 0.71
India-1 buffalo	1.08	0.47	**0.02**	**2.94**	1.17, 7.40
India-1 cattle	−0.13	0.3	0.66	0.88	0.49, 1.58
India-2 cattle	(ref)				
Vietnam buffalo	−0.10	0.42	0.81	0.90	0.40, 2.06
Vietnam cattle	0.68	0.25	**0.007**	**1.97**	1.21, 3.22

* *Coef, Model coefficient*.

** *Std. Err, standard error*.

*Significant values are indicated in bold*.

For all groups combined, the overall probability of persistent infection in seropositive animals predicted using the GLMM final model was 80.38% (range: 75.21–91.05%) at 6 months after an outbreak, 32.08% (range: 24.23–51.75%) at 12 months post-outbreak, 5.17% (range: 3.26–10.16%) at 18 months post-outbreak, and at 24 months post-outbreak was 0.6% (range: 0.35–1.18%) (Table 2, Figure 4C).

## Discussion

Persistent infection with foot-and-mouth disease virus is a challenge for FMD control and eradication in endemic regions. Similarly, FMD-free regions must consider the existence of carriers when responding to incursions. Additionally, neoteric subclinical infection is indistinguishable from persistence under field conditions, and may pose a greater threat of transmission (5, 42). Although the role of persistently infected animals in FMDV epidemiology remains controversial, persistently infected animals are known to carry virus in a form that is directly infectious to susceptible animals (15). This is consistent with the demonstrated very low, but non-zero, quantitative risk of transmission (13, 14). Because of this, global FMD control policies must consider persistent as well as acute infection. Quantitative estimates of the probability of persistent infection at specified times post-outbreak may provide a tool to more accurately assess the potential risks posed by persistently infected animals, which will help to guide control efforts. The current study compared two statistical modeling approaches, a survival analysis model (AFT) and generalized linear model (GLMM), for estimating the probability of persistent infection, while evaluating the benefits of meta-analytical approaches to leverage primary datasets that span a wider range of conditions including study design and duration, virus-specific factors, host-specific factors, and environmental factors.

In the by-country analyses of the Vietnam primary study, the AFT and GLMM models generated similar assessment of the impact of species on the duration of persistent infection. In both modeling approaches, the duration of persistent infection did not differ significantly between cattle and buffalo. Similarly, a previous study reported that the odds of persistent infection did not differ significantly between dairy cattle and buffalo, although the odds were significantly higher for beef cattle being carriers in that study (27). It is possible that either distinct host genetics or the different management practices for beef vs. dairy cattle influence viral extinction. Overall, our results indicate that species is not a significant factor affecting persistent infection in cattle and buffalo in Vietnam.

For the Vietnam analyses, the predicted probabilities of persistent infection among seropositive animals were similar between the modeling approaches at 18 and 24 months post-outbreak. Both models predicted <5% probability of persistent infection at 24 months post-outbreak. In contrast, a previous study in Vietnam estimated that the mean duration of persistent infection was 27 months (14). This discrepancy may be partially explained by the small sample size (*n* = 10) and large uncertainty (±6 months) in the outbreak dates used in the previous study. The sample collection period may also influence the estimated duration of persistent infection. Due to the logistics of field sampling, we were unable to collect samples earlier than 14 months post-outbreak. Although model predictions were similar at later timepoints, predictions were widely different at 6 and 12 months post-outbreak, likely due to the lack of data at these timepoints and differences in assumptions between models. These results highlight the need for robust datasets to develop accurate models. Further studies in Vietnam or other endemic settings should include earlier timepoints to more accurately describe the dynamics of the extinction of the FMDV carrier state.

In contrast to the Vietnam study, the models differed in their estimates of duration and significance for the by-country analyses of the India studies. In the AFT model, the duration of persistent infection did not differ for India-1 buffalo compared to India-2 cattle, whereas the GLMM estimated a significantly longer duration for India-1 buffalo. Similarly, a previous analysis of the India-1 primary study reported that a higher proportion of buffalo were persistently infected compared to cattle; however the difference was not significant in that study (34). Although both models herein estimated a shorter duration of persistent infection for India-1 cattle compared to India-2 cattle, the difference was only significant in the AFT model. Differences between model results for India-1 buffalo may be due to the relatively small (*n* = 17) number of buffalo included in the study. Differences in results may also be due to differences in data handling between the two models. The input unit for the AFT was animal, whereas the input unit for GLMM was sample, and the difference in number of samples between the India-1 and India-2 studies was much greater than the difference in number of animals.

Despite the differences between models, the predicted probabilities of persistent infection among identified carriers were largely similar between the models at 6 and 12 months post-infection for the India analyses. The predicted probabilities of persistent infection at 12 months post-outbreak were consistent with previous analyses of the India data, which used the Kaplan-Meier estimator to estimate the duration of persistent infection. Previously, 14% of cattle were reported to be persistently infected at 10.5 months post-outbreak in the India-1 study (28), and the average duration of persistent infection was 13 months in the India-2 study (19). Similar to the Vietnam analyses, model predictions were more divergent at timepoints beyond the sample collection period for the India studies (18 and 24 months post-outbreak).

Although model predictions were similar in the by-country analyses for the timepoints within the collection period for the studies, predictions were widely different when the models extrapolated beyond the range of the data. These differences in predictions are due to differences in data handling between the models. AFT utilizes a single data point for each animal—the interval in which the animal cleared the infection—and assumes every animal is persistently infected until that interval. The model assumes the initial proportion of persistently infected animals is 100% and can only decrease over time (43). Additionally, AFT models are constrained by the distribution specified when building the model. In contrast, GLMM utilizes multiple data points per animal—each individual sample—and animals may have intermittent negative samples due to imperfect tests for detection of FMDV in OPF (32). The model does not assume an initial proportion of persistently infected animals, and the proportion is allowed to vary over time (43). These differences in the models result in more divergent predictions outside the data range, particularly when a limited range of timepoints is used to build the models. To overcome the limited ranges of timepoints in the individual studies in our analyses, we combined all three studies in an additional analysis to further assess the dynamics of FMDV persistent infection.

In the combined meta-analyses of all three studies, the AFT and GLMM models had the same direction of change compared to India-2 cattle for all groups except India-1 buffalo; however the significance of the change was different for all groups except Vietnam buffalo. These results suggest the trends of the associations between study/species group and duration of persistence are reliable; however the significance of the associations should be interpreted cautiously and in consideration of the model used in the analysis. As expected, model predictions were more similar for the combined analyses than for the analyses by country, highlighting the benefits of increased sample size and range of collection times in the combined analyses. However, based on data availability, the current analyses were limited to studies in Asia and included mostly vaccinated animals as well as relatively low diversity of FMDV strains. Vaccination does not protect against persistent infection, and previous studies have shown no difference in the proportion of carriers among vaccinated and naïve animals (8, 44, 45). Therefore, vaccination likely does not affect the duration of persistent infection, however this should be investigated in future studies. Due to the potential variability across viral strains, environmental conditions, and host genetic backgrounds, future studies should assess FMDV persistent infection across a wider geographical area and virus diversity.

Additionally, both models assume no reintroductions or incursions of novel FMDVs during the primary studies. This assumption was based upon official records from DAH, Vietnam and herd health records for the India studies that indicated no new outbreaks of FMD occurred in the herds contributing to the primary studies (19, 28). Additionally, viral sequences obtained from consecutive samples from a subset of the animals indicated these animals were not re-infected with a different FMDV strain (27, 34). Neoteric subclinical infection (5) was largely ruled out by lack of detection of sequences of novel strains within the geotemporal space of the primary studies (27, 34), but cannot be completely excluded since sequence data could not be obtained from every sample from which FMDV RNA was detected.

In the meta-analyses, the AFT and GLMM models predicted >98% and 75–91% probability of persistent infection in seropositive animals at 6 months post-outbreak, respectively. In contrast, a previous meta-analysis of four experimental studies reported only 52% of animals were persistently infected at 6 months post-outbreak (13). The longer duration of persistent infection in the current study may reflect differences in infection dynamics between natural and experimental conditions, including controlled exposure and small sample sizes in experimental studies. Model predictions at 12 months post-outbreak were consistent with a previous field study which reported 20% of animals were persistently infected 12 months post-outbreak (46). In contrast, a previous probability analysis reported substantially lower (0.7%) probability of persistent infection at 12 months post-outbreak (25). The wide discrepancy between this previous analysis and the current analyses may be due in part to differences in study design. For example, the three primary studies in the current analyses were longitudinal studies, specifically intended to monitor viral extinction in carriers, and included only animals with previous exposure to FMDV (Vietnam) or identified carriers (India). By contrast, the Bronsvoort et al. (25) study used a cross-sectional study design and included unexposed animals as well as animals previously exposed to FMDV. Furthermore, the previous analysis was also limited to a single study setting, with accompanying limitations on sampling times post-outbreak and host, viral, and environmental factors. The current study used detection of viral RNA in OPF to determine carrier status. RNA detection by rRT-PCR may be more sensitive than virus isolation (VI) under some circumstances (47, 48), which would result in a higher apparent prevalence of carriers in these analyses. However, in a previous analysis of the India-1 study herein, the duration of persistent infection was not different as determined by rRT-PCR or VI (34). Similarly, other experimental studies have reported comparable sensitivities for rRT-PCR and VI (8), suggesting that viral RNA detection similarly reflects the true carrier status of an animal. The current study attempted to improve upon previous analyses of the extinction of the FMDV carrier state by combining data from three distinct primary studies to develop mathematical models that represent the dynamics of persistent infection, and then use the models to predict the probability of persistent infection at specified times post-outbreak. These analyses provide a more tailored approach to the development of control measures to minimize the risk posed by persistently infected animals.

Overall, the two models produced similar predictions in the combined analyses, suggesting that either model may be satisfactory for describing the dynamics of FMDV carrier state extinction. Researchers should consider which model is more appropriate for a particular study based on the study design, data structure, and whether model assumptions are biologically appropriate. Additionally, results should be interpreted in consideration of the model used for analyses. Our results suggest that when only cross-sectional data are available, a GLMM approach may be suitable to model the probability of FMDV persistent infection in the study population. Cross-sectional studies are less expensive and can be completed faster than longitudinal studies, offering advantages in risk assessment relating to persistently infected animals in an endemic population. However, detection of virus in OPF is inconsistent (6, 14, 19, 49), and cross-sectional studies may, therefore, underestimate the proportion of persistently infected animals at any given time post-outbreak. Additionally, a wide range of times post-outbreak is needed to more accurately model persistent infection dynamics, and repeated cross-sections may be needed to achieve this. As demonstrated in the current study, meta-analysis of several longitudinal studies can overcome some limitations of individual studies, such as small sample size and limited sampling frequency, while providing a more robust view of viral dynamics within animals over time. Furthermore, this approach can help researchers and disease control experts better understand how persistent infection varies across populations.

## Conclusion

FMDV persistent infection causes a substantial economic burden on endemic countries due to trade restrictions, which have traditionally treated persistent infection as a binary state (present/absent) with a fixed duration. However, persistent infection is a dynamic process, and statistical models can be useful to assess the decreasing probability of persistent infection at increasing times post-outbreak. Additionally, meta-analysis reduces the impact of limitations in individual studies. In the current study, the AFT and GLMM models predicted similar probabilities of persistent infection at 18 and 24 months post-outbreak, while the probability of detection of persistent infection was higher using the AFT model at 6 and 12 months post-outbreak. Because it may over-estimate probabilities at earlier timepoints, the AFT model is the more cautious approach for designing policies to reduce or eliminate the potential risk presented by persistently infected animals following an FMD outbreak. Additional studies with larger sample sizes and expanded meta-analyses, including more primary studies, are likely to provide more nuance and depth to our understanding of this dynamic process, leading to an improved understanding of FMDV persistent infection after outbreaks and how to predict and manage this disease state.

## Data Availability Statement

The datasets analyzed during the current study are available from the authors upon reasonable request.

## Ethics Statement

Ethical review and approval was not required for the animal study because the work described herein was performed by federal staff of the Department of Animal Health, Ministry of Agriculture and Rural Development, Government of Vietnam or the Directorate of Foot and Mouth Disease, Indian Council of Agricultural Research, Ministry of Agriculture, Government of India. The work occurred and the animals were maintained within facilities that were owned, maintained, or overseen by these divisions of the federal governments; thus, no permits or approvals were required. All cases described herein occurred spontaneously in domestic cattle or buffalo with no experimentation, inoculation, or treatment of live animals.

## Author Contributions

MB performed the statistical analysis and drafted the manuscript. SY contributed to the statistical analysis, and helped to draft the manuscript. AD participated in the design and coordination, and helped to draft the manuscript. CS contributed to study design of primary studies and drafting and revising the manuscript. JA conceived the study, participated in the design and coordination, and helped to draft the manuscript. All authors read and approved of the final manuscript.

## Conflict of Interest

The authors declare that the research was conducted in the absence of any commercial or financial relationships that could be construed as a potential conflict of interest.
